# Supplementary material for: Asynchronous magnetic resonance elastography: Shear wave speed reconstruction using noise correlation of incoherent waves
Source: Magn Reson Med. 2022 Oct 27;89(3):990–1001. doi: 10.1002/mrm.29502 (PMC9792433; doi:10.1002/mrm.29502)
Supplement: Supplementary file 1 — DATA S1 MATLAB code used to generate the numerical simulations of Figure 1 [file MRM-89-990-s002.zip › k-Wave/helpfiles/example_us_beam_patterns.html]

Simulating Ultrasound Beam Patterns Example (k-Wave)


# Simulating Ultrasound Beam Patterns Example

This example shows how the nonlinear beam pattern from an ultrasound transducer can be modelled. It builds on the Defining An Ultrasound Transducer and Simulating Transducer Field Patterns examples.

- Open the file in the MATLAB Editor
- Run the file in MATLAB

## Contents

- Simulating the total beam pattern
- Simulating harmonic beam patterns using the recorded sensor data
- Extending the simulations

## Simulating the total beam pattern

Once a transducer has been created (see Defining An Ultrasound Transducer), a map of the resulting rms or maximum pressure within the medium can be produced (this distribution is typically called a *beam pattern*). This is done using a binary sensor mask which covers the plane of interest. For example, to compute the beam pattern in the x-y plane that dissects the transducer, the sensor mask should be defined as shown below (set `MASK_PLANE = 'xy';` within the example m-file).

```
% define a sensor mask through the central plane of the transducer
sensor.mask = zeros(Nx, Ny, Nz);
sensor.mask(:, :, Nz/2) = 1;
```

If only the total beam pattern is required (rather than the beam pattern at particular frequencies), this can be produced without having to store the time series at each sensor point by setting `sensor.record` to `{'p_rms', 'p_max'}`. With this option, at each time step k-Wave only updates the maximum and root-mean-squared (RMS) values of the pressure at each sensor element. This can significantly reduce the memory requirements for storing the sensor data, particularly if sensor masks with large numbers of active elements are used.

```
% set the record mode such that only the rms and peak values are stored
sensor.record = {'p_rms', 'p_max'};
```

After the simulation has run, the resulting sensor data is returned as a structure with the fields `sensor_data.p_max` and `sensor_data.p_rms`. These correspond to the maximum and RMS of the pressure field at each sensor position. Because the time history is not stored, these are indexed as `sensor_data.p_max(sensor_position)`. This data must be reshaped to the correct dimensions before display.

```
% reshape the returned rms and max fields to their original position
sensor_data.p_rms = reshape(sensor_data.p_rms, [Nx, Ny]);
sensor_data.p_max = reshape(sensor_data.p_max, [Nx, Ny]);
```

A plot of the beam pattern produced using the maximum pressure in the x-y and x-z planes is shown below. Note, in this example the number of active sensor elements is set to 32. To allow the the size of the computational domain to be reduced (and thus speed up the simulation) `transducer.number_elements` is also set to 32.


## Simulating harmonic beam patterns using the recorded sensor data

If the harmonic beam pattern is of interest (the beam pattern produced at different frequencies), the time series at each sensor position must be stored, and then analysed after the simulation has completed (set `USE_STATISTICS = false;` within the example m-file). To reduce the memory consumed as a result of storing the time history over a complete plane (which can become significant for larger simulations), the sensor data can be incrementally streamed to disk by setting the optional input `'StreamToDisk'` to `true`. Alternatively, `'StreamToDisk'` can be set to the number of time steps that are stored before saving the data to disk. This is useful if running simulations on GPUs with limited amounts of memory.

After the simulation is complete, there are several processing steps required to produce the beam pattern. These are shown in the code snippet below. Here `j` corresponds to the second axis of interest. This will by the y-axis if `MASK_PLANE` is set to `'xy'` within the example m-file, or the z-axis if this is set to `'xz'`. The values of `beam_pattern_f1` and `beam_pattern_f2` correspond to the relative spectral amplitudes at the fundamental and second harmonic frequencies of the input signal used to drive the transducer (0.5 MHz in this example).

```
% reshape the sensor data to its original position so that it can be
% indexed as sensor_data(x, j, t)
sensor_data = reshape(sensor_data, [Nx, Nj, kgrid.Nt]);

% compute the amplitude spectrum
[freq, amp_spect] = spect(sensor_data, 1/kgrid.dt, 'Dim', 3);

% compute the index at which the source frequency and its harmonics occur
[f1_value, f1_index] = findClosest(freq, tone_burst_freq);
[f2_value, f2_index] = findClosest(freq, 2 * tone_burst_freq);

% extract the amplitude at the source frequency and store
beam_pattern_f1 = amp_spect(:, :, f1_index);

% extract the amplitude at the second harmonic and store
beam_pattern_f2 = amp_spect(:, :, f2_index);       

% extract the integral of the total amplitude spectrum
beam_pattern_total = sum(amp_spect, 3);
```

A plot of the beam pattern at the fundamental frequency and the second harmonic are shown below.


These distributions can also be used to analyse the lobe widths of the fundamental frequency and harmonics at the transducer focus. An example of this is given below.


## Extending the simulations

This example can be used as a framework for simulating the beam patterns produced by a wide range of linear transducers. For example, the number of grid points in the computational grid could be increased to allow higher transmit frequencies to be studied. Similarly, the effect of apodization on the beam characteristics could be investigated by changing the value of `transducer.transmit_apodization` to one of the inputs accepted by `getWin` (or to a custom apodization). As the simulations are performed in 3D, both on-axis and off-axis responses can be visualised (change `MASK_PLANE` to `'xz'`). Beam patterns in heterogeneous media can also be generated by assigning heterogeneous medium parameters to `medium.sound_speed` and `medium.density`.
